# A Case Study: Preserved Nipple-Areolar Complex Vascularity in High-Risk Secondary Reduction Mammaplasty Using Single-Stage Suction-Assisted Lipectomy With Dermal Mastopexy

**DOI:** 10.7759/cureus.43247

**Published:** 2023-08-09

**Authors:** Isabella Ho, Tina Moon, Daniel Driscoll

**Affiliations:** 1 Plastic and Reconstructive Surgery, Tufts Medical Center, Boston, USA

**Keywords:** suction-assisted lipectomy, breast reconstruction, dermal mastopexy, breast reduction, reduction mammaplasty

## Abstract

Repeat surgery is known to increase risk of several surgical complications, including compromise to the blood supply of/surrounding the surgical site. As such, we offer an alternative to the use of a standard breast reduction technique in the case of a re-do reduction, pursued with a goal of maintaining the blood supply to the nipple-areola complex. When compared to traditional reduction mammoplasty, suction-assisted lipectomy with dermal mastopexy has been demonstrated to be a highly effective technique in protecting the vascularity of the nipple-areola complex in repeat breast reductions. We describe a successful utilization of this technique for a high-risk patient with active tobacco use undergoing secondary reduction mammoplasty.

## Introduction

It is well-known in plastic surgery that cigarette smoking and other forms of nicotine intake are associated with increased wound healing complications [1-6]. A retrospective analysis of 13,503 patients who underwent reduction mammoplasty found that smokers had the highest likelihood of any wound complication compared to non-smokers [7]. In a randomized controlled trial of 67 patients, Srinivasaiah demonstrated a 37% difference in complication rates when comparing current smokers with non-smokers [8]. Despite recommendations of abstaining from smoking in the pre-operative period, Cruz et al. found that former smokers had significantly higher rates of surgical site infections, wound dehiscence, fat necrosis, nipple necrosis, and re-operation/revision compared to never smokers [9].

Complication rates are known to be higher in repeat reduction mammoplasty compared to initial reduction mammoplasty [10]. A systematic review of 244 patients requiring repeat breast reductions found a major complication rate of 2.5% and minor complication rate of 9.4% [11]. Major complications were defined as nipple-areolar complex (NAC) necrosis/loss, congestion, or major seroma/abscess. Minor complications were defined as delayed wound healing, nipple sensitivity, mild fat necrosis, minor necrosis of areolar edge, dog ear, or small hematoma. It is with these cases in mind that we would like to share and discuss the success of a particularly high-risk secondary breast reduction.

## Case presentation

**Figure 1:**
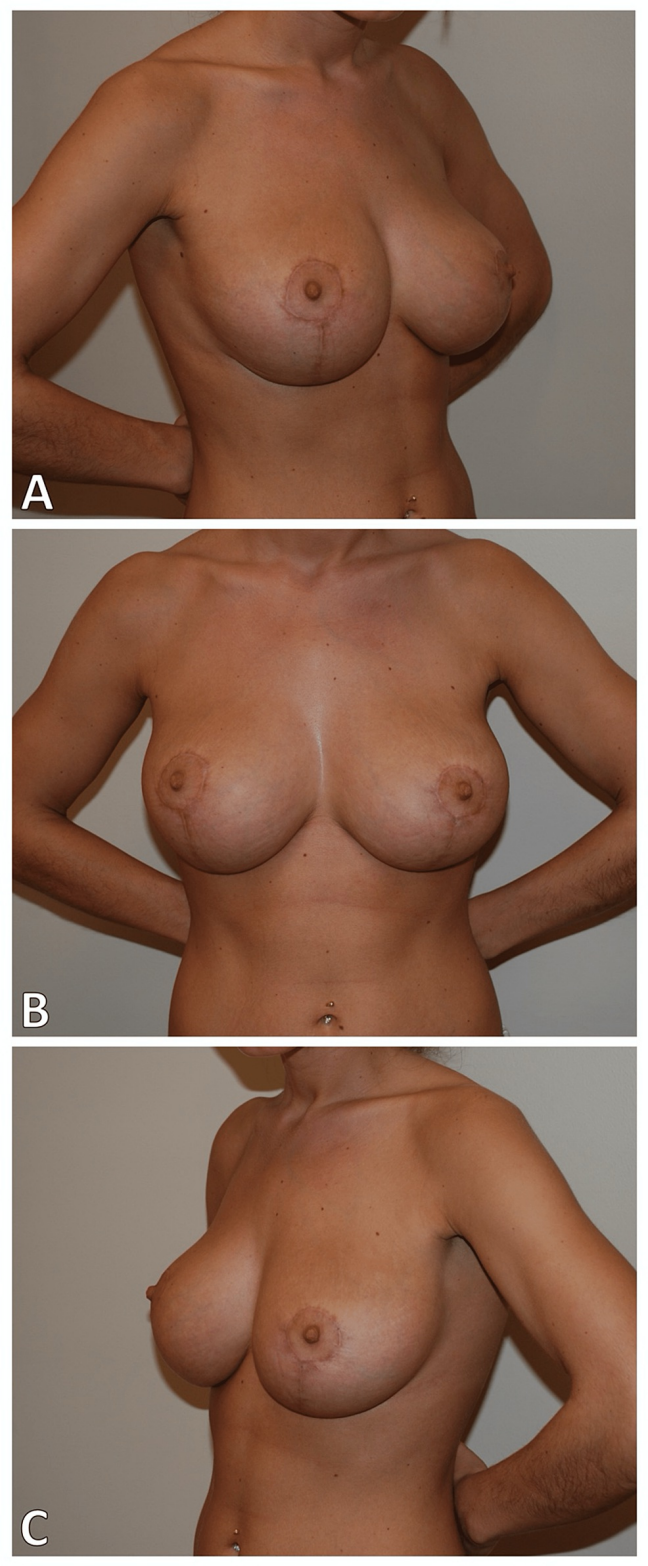
Surgical result of prior breast reduction in 2009 (A) Right lateral view (B) Medial view (C) Left lateral view

**Figure 2:**
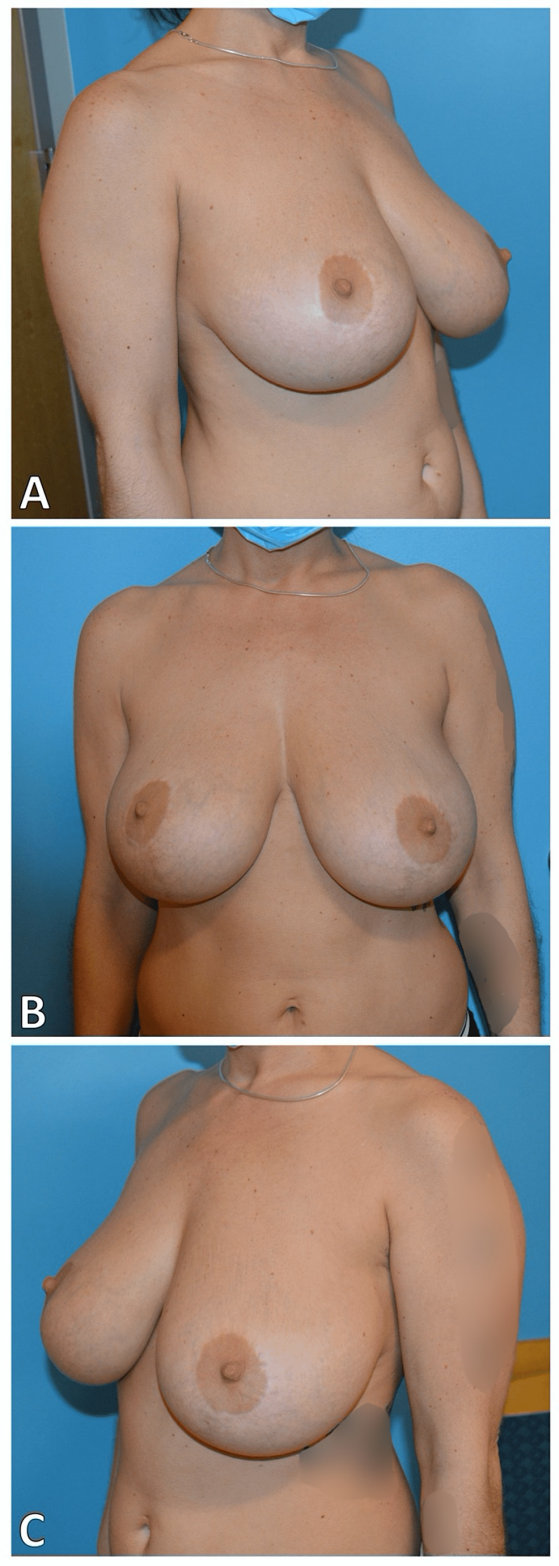
Preoperative view, 13 years status post prior breast reduction (A) Right lateral view (B) Medial view (C) Left lateral view

A 35-year-old female underwent a re-do bilateral breast reduction with positive surgical result. The patient had previously undergone reduction mammoplasty in 2009 (Figure 1). However, given post-surgical weight gain and subsequent breast growth, the patient suffered recurrence of her back, neck, and shoulder pain. As such, she elected to undergo a repeat procedure (Figure 2).

**Figure 3:**
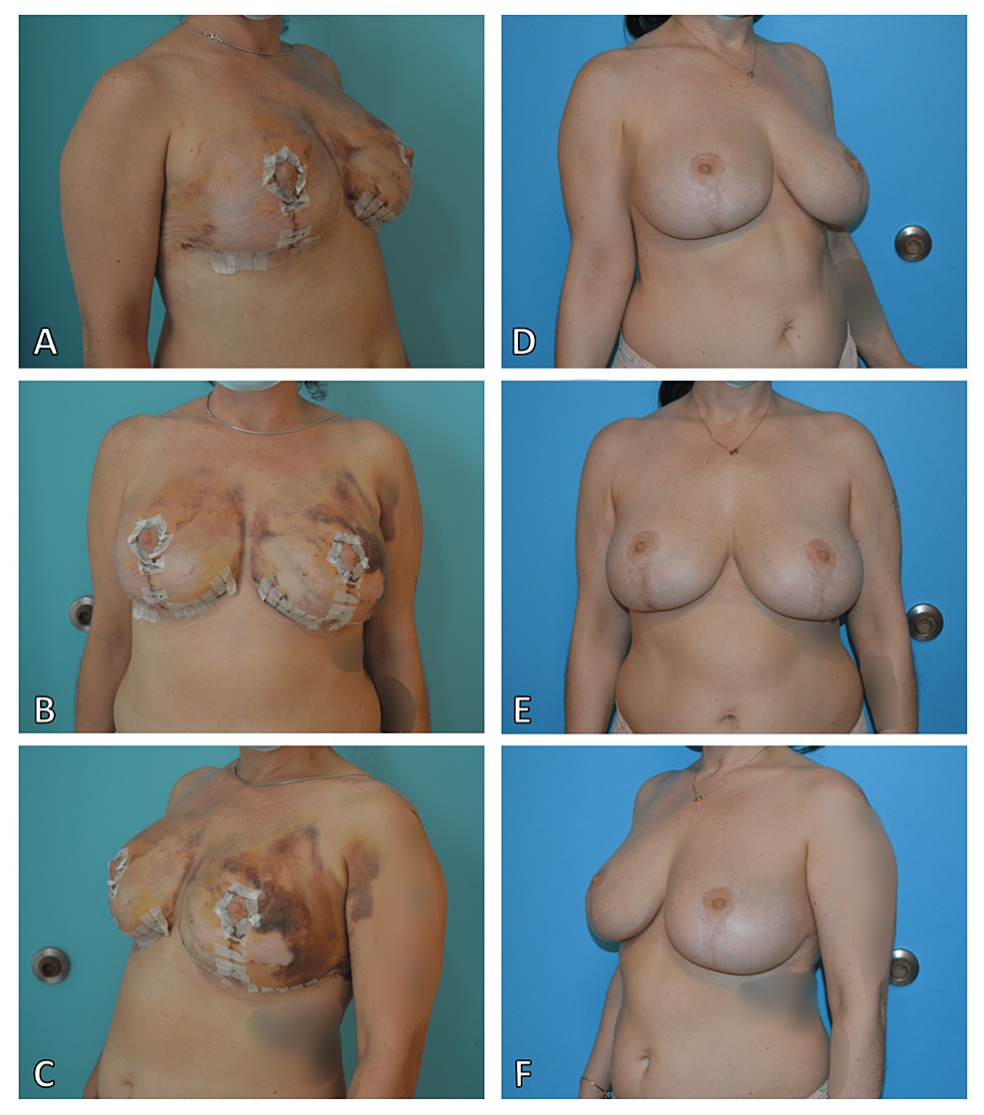
Post-operative follow-up (A) One-week postoperative, right lateral view. (B) Medial view. (C) Left lateral view. (D) Six-month postoperative, right lateral view. (E) Medial view. (F) Left lateral view.

Due to the risk of complications in repeated reduction mammaplasty, we chose to pursue suction-assisted lipectomy with dermal mastopexy, rather than traditional reduction mammoplasty [11]. 500cc of tumescent with TXA were injected to each breast during the operation, and 1100cc were aspirated from each side for the reduction. As documented in "Single Stage Suction-Assisted Lipectomy with Dermal Mastopexy: An Alternative Procedure in Repeat Reduction Mammaplasty with Questionable Nipple-Areola Complex Vascularity", this technique allows for greater preservation of the nipple-areola complex vasculature and sensation without the risks of compromising blood supply in the settings of unknown prior pedicles [12]. On follow-up visits, it was discovered that the patient had been smoking cigarettes and using nicotine-based vapes daily, both prior to her surgery and throughout her recovery. This was not known pre-operatively; as the patient had denied the use of any nicotine-containing products in consult, and no cotinine test was obtained. On discovery of her smoking/vape use in follow-up, she was repeatedly counseled on smoking risks and cessation. While the patient declined to adjust her nicotine intake, she was consistently noted to be healing well throughout all subsequent follow-up visits, without seroma/hematoma, wound dehiscence, fat necrosis, signs of infection, disturbances in nipple healing/sensation, or other such complications (Figure 3).

## Discussion

The patient’s surgical result is remarkable given the presence of two significant, co-existing risk factors. As found by Cruz on review of reduction mammoplasty results in 298 women, those with a history of smoking alone were at statistically increased risk for post-surgical complications, even when abstaining from nicotine intake for a minimum of four weeks prior to surgery. Of particular interest to our case, rates of nipple necrosis were found to increase from 2% in never smokers to 15% in prior smokers [9]. A review of breast reduction results conducted by Patel et al. found three of eight repeat reduction mammaplasty patients experienced a major complication (i.e. nipple necrosis, abscess, and seroma). This 37.5% complication rate was noted to be significantly higher than the 4-26% complication rate associated with 882 primary breast reductions performed within the same health system [13]. Similarly, Losee et al.’s review of secondary reduction mammaplasty cases revealed complications in four out of 10 patients [14]. Given our patient’s continued tobacco use, in addition to the known increased complication rates associated with repeat breast reduction, a complication in her case would have been unsurprising [10,13,14]. However, her immediate postoperative period was notable only for ecchymosis of the bilateral breasts, which resolved without issue or need for intervention. Her healing course was negative for issues related to compromised blood supply, despite her prior breast surgery and the vasoconstriction associated with nicotine use. As such, her results demonstrate the potential to mitigate complication through this conservative surgical technique.

## Conclusions

This patient’s case speaks to the effectiveness of suction-assisted lipectomy with dermal mastopexy for repeat reduction mammoplasties as a method of both protecting blood supply and reducing complication rates. Cessation of smoking/nicotine intake pre-operatively and post-operatively should continue to be advised. As previously discussed, the literature has repeatedly demonstrated a higher likelihood of poor healing associated with nicotine. However, with this patient, the effects of nicotine-based vasoconstriction were successfully mitigated, indicating the potential benefit of this particular technique for high-risk patient populations. 
